# A Rare Coexistence: Drug Induced Hepatitis and Meningitis in Association With Ibuprofen

**DOI:** 10.4021/jocmr1280w

**Published:** 2013-04-23

**Authors:** Suresh Kumar Nayudu, Shilpa Kavuturu, Masooma Niazi, Myrta Daniel, Anil Dev, Kavitha Kumbum

**Affiliations:** a Division of Gastroenterology and Hepatology, Bronx Lebanon Hospital Center, Affiliated with Albert Einstein College of Medicine, Yeshiva University, Bronx, New York, USA; b Department of Medicine, Bronx Lebanon Hospital Center, Affiliated with Albert Einstein College of Medicine, Yeshiva University, Bronx, New York, USA; c Department of Pathology, Bronx Lebanon Hospital Center, Affiliated with Albert Einstein College of Medicine, Yeshiva University, Bronx, New York, USA

**Keywords:** Ibuprofen, Hepatitis, Meningitis

## Abstract

Ibuprofen, a commonly used NSAID is reported to be associated with drug induced liver injury. Ibuprofen is also known to be associated with drug-induced meningitis especially in patients with connective tissue disorders. However presentation of hepatitis and meningitis in association with Ibuprofen use in the same individual has never been reported. We present a case of young woman who developed abnormal liver chemistries and neurological symptoms while on Ibuprofen. Her liver biopsy findings were suggestive of drug induced liver injury and cerebrospinal fluid analysis was suggestive of aseptic meningitis. Clinical and biochemical improvement was noted on cessation of Ibuprofen.

## Introduction

Nonsteroidal anti-inflammatory drugs (NSAIDS) are known to be associated with many adverse drug reactions involving gastrointestinal tract and liver [1, 2]. Ibuprofen, a commonly used NSAID is reported to be associated with drug induced liver injury [3, 4]. Ibuprofen is also known to be associated with drug-induced meningitis especially in patients with connective tissue disorders [5, 6]. However, simultaneous presentation of hepatitis and meningitis in association with Ibuprofen use in the same individual has never been reported. We present a case of young woman who developed abnormal liver chemistries and neurological symptoms, which resolved after cessation of Ibuprofen.

## Case Report

A 38-year-old African American woman came to emergency room with generalized body aches. She also reported three episodes of vomiting containing clear liquid and subjective fever. She denied respiratory, cardiovascular or neurological symptoms at the time of initial evaluation. Her past medical history was negative for any medical conditions. Her surgical history included right knee arthroscopy few years back. She denied tobacco, alcohol or recreational drug use. She denied any allergies to medications in the past.

In the emergency room she was found to have temperature of 100 degrees of Fahrenheit, heart rate of 72 per minute, systolic blood pressure of 164 and diastolic blood pressure of 64 millimeters of mercury respectively. On examination she was a well built woman without any distress. Her abdominal examination did not reveal any tenderness, organomegaly or clinically detectable free fluid. Her cardiovascular, respiratory and neurological examination was within normal limits. Her laboratory and imaging tests revealed abnormal liver chemistries (Table 1) and dilated common bile duct (CBD) of 8 millimeters without any evidence of cholelithiasis or choledocholithiasis. She was hydrated with intravenous fluids and treated symptomatically with Ibuprofen 800 mg for body aches as needed. She was admitted to medical floor for further work up and management.

**Table 1:** Liver Chemistries

	Day 1	Day 2	Day 10	2 weeks	3 months
Total protein^1^	8.6	7.6	6.9	7.5	8.3
Albumin^1^	4.2	3.7	3.3	3.8	4.3
Alanine aminotransferase^2^	249	464	59	28	10
Aspartate aminotransferase^2^	201	383	24	17	14
Alkaline phosphatase^2^	31	36	47	40	40
Total bilirubin^3^	0.4	0.4	0.4	0.8	0.3
Direct bilirubin^3^	0.1	0.1	0.2	0.2	0.1

1.grams/dL, 2. Units/liter, 3. Milligrams/dL.

On day 2, gastroenterology evaluation was requested for abnormal liver chemistries. She did not report any further episodes of vomiting and her body aches slightly improved with ibuprofen. On further interviewing it was noted that recently she fell and injured her left knee. She was evaluated by orthopedician and noted to have anterior cruciate ligament, medial meniscus and medial collateral ligament tears on magnetic resonance imaging (MRI). She mentioned that for last one week she had been taking over-the-counter Ibuprofen 600 mg tablets as needed for her knee pain. Extensive laboratory work up aimed to diagnose viral, metabolic and autoimmune liver diseases was requested. Magnetic resonance cholangiopancreatography (MRCP) was requested to rule out biliary obstruction, as there was CBD dilatation noted on abdominal sonogram. It was also recommended to stop Ibuprofen as initial presumptive diagnoses included drug induced liver injury.

Her liver chemistries showed alaninie aminotransferase (ALT) of 249 units/liter, aspartate aminotransferase (AST) of 201 units/liter, alkaline phosphatase (ALP) of 31 units/liter with normal total protein, albumin and bilirubin levels. Her other significant laboratory results include platelet count of 139,000/micro liter and potassium levels of 3 milliequivalents/liter. She was immune to hepatitis B and tested negative for hepatitis A, hepatitis C and human immunodeficiency (HIV) viruses. Her transferrin saturation was 16% and ceruloplasmin levels were within normal limits excluding the possibility of hemochromatosis and Wilson disease respectively. Autoimmune markers including anti-nuclear antibody (ANA), anti-mitochondrial antibody were negative except for anti-smooth muscle antibody titers of 1:20, which was not clinically significant. MRCP was done which showed dilated common bile duct of 9 millimeters without any evidence of choledocholitiasis or obstructing lesion.

**Figure 1:**
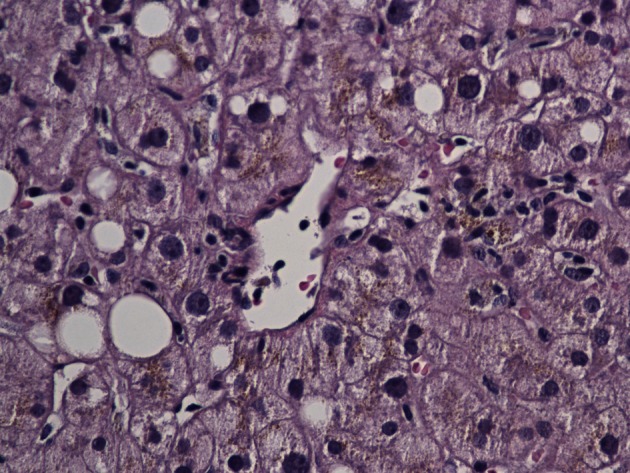
Liver biopsy specimen showing lobular hepatitis.

**Figure 2:**
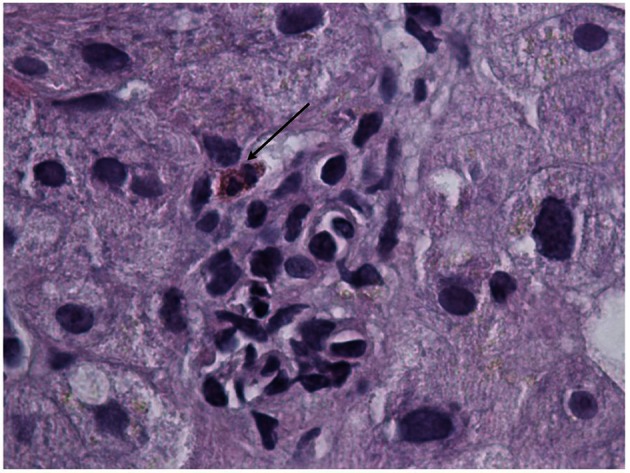
Liver biopsy specimen revealing eosinophilic infiltrate.

On day 3, she became lethargic without any focal neurological deficit and was transferred to medical intensive care unit (MICU) for close monitoring. Computer tomography (CT) and MRI of brain ruled out acute intracranial pathology and lumbar puncture with cerebrospinal fluid (CSF) analysis was performed. Her CSF analysis showed elevated protein, normal glucose and lymphocytic pleocytosis. CSF was tested negative for common bacterial and viral pathology. Her blood and urine were tested negative for common toxicological agents. Her mental status gradually improved after cessation of Ibuprofen and transferred back to medical floor. She underwent CT guided liver biopsy which was uneventful. Liver biopsy (Fig. 1, 2) was reported to have mild lobular hepatitis with eosinophilic infiltrate suggestive of drug induced liver injury.

Her hospital course was significant for an episode of fever associated with leucocytosis. Complete septic work up including blood, urine cultures and x-ray of chest were done. She received broad-spectrum antibiotics and improved without any further febrile episodes. Her x-ray was negative for any lung pathology and body fluid cultures were negative. During this period she also developed acute renal failure with blood urea nitrogen of 32 milligrams/dL and creatinine of 3 milligrams/dL, which improved with conservative management. Subsequently her clinical condition improved and all her laboratory values including liver chemistries, electrolytes and platelets were normalized leading to her discharge from hospital.

On further follow up in clinic, she was asymptomatic and her all laboratory tests were within normal limits. She underwent endoscopic ultrasound (EUS) which did not reveal any CBD stone or obstructive lesion. She was subsequently evaluated by orhtopedician and underwent arthroscopic intervention for her knee injury.

## Discussion

NSAIDS are known to be associated with various adverse events especially involving gastrointestinal tract and liver [1, 2]. Ibuprofen, a commonly used NDSAID is known to cause various gastrointestinal adverse effects but with lesser frequency [2]. Drug induced liver injury in association with Ibuprofen was first reported in 1977. Subsequently it has been reported in various clinical scenarios where therapeutic doses of ibuprofen were associated with fatty liver, transaminitis and cholestatic hepatitis [7-10]. There have been rare occasions where ibuprofen associated liver injury resulted in serious consequences requiring liver transplantation [3, 4, 11].

Drug induced meningitis or aseptic meningitis in association with Ibuprofen is mainly characterized by clinical features of meningoencephalitis with elevated protein, normal glucose levels and neutrophilic or lymphocytic pleocytosis in CSF, but without any evidence of microbial involvement [12, 13]. In majority of occasion’s Ibuprofen induced aseptic meningitis is reported in individuals with connective tissue disorders [5, 6, 14, 15]. There are variations in presentation including eosinophilic meningitis [16], involving eyes leading to iridocyclitis [17]. There are also reports that meningitis can recur after re-challenging with Ibuprofen or a different NSAID [12, 18, 19].

Our case has unique features of absence of any known rheumatologic or connective tissue disorders and simultaneous presentation of drug induced meningitis and hepatitis. Our patient was taking Ibuprofen prior to admission to the hospital, which was continued during initial days of her stay in hospital to control pain. She developed neurological symptoms and work up was negative for any bacterial or viral etiology. Her liver chemistries and neurological symptoms improved after cessation of ibuprofen supporting diagnosis of drug induced mechanism. Physicians should be cautious when prescribing NSAIDS due to their safety profile and educate patients accordingly. Physicians should also make themselves familiar with complete adverse reaction profile of medications which guide them in timely diagnosis and management of complications as most of the complications associated with Ibuprofen are reversible with the cessation of medication [10, 12, 20].
